# Predicting bacterial vaginosis incidence using artificial neural networks

**DOI:** 10.1371/journal.pcbi.1014768

**Published:** 2026-09-15

**Authors:** Jacob H. Elnaggar, John W. Lammons, Caleb M. Ardizzone, Kristal J. Aaron, Clayton Jacobs, Keonte J. Graves, Sheridan D. George, Megan Amerson-Brown, Meng Luo, Ashutosh Tamhane, Paweł Łaniewski, Alison J. Quayle, Melissa M. Herbst-Kralovetz, Nuno Cerca, Christina A. Muzny, Christopher M. Taylor

**Affiliations:** 1 Department of Microbiology, Immunology, and Parasitology, Louisiana State University Health Sciences Center, New Orleans, Louisiana, United States of America; 2 Division of Infectious Diseases, Department of Medicine, Indiana University School of Medicine, Indianapolis, Indiana, United States of America; 3 Division of Infectious Diseases, Department of Medicine, University of Alabama at Birmingham, Birmingham, Alabama, United States of America; 4 Division of Laboratory Science, Department of Pathology, University of Alabama at Birmingham, Birmingham, Alabama, United States of America; 5 Division of Nephrology, Department of Medicine, University of Alabama at Birmingham, Birmingham, Alabama, United States of America; 6 Department of Basic Medical Sciences, College of Medicine-Phoenix, University of Arizona, Phoenix, Arizona, United States of America; 7 Centre of Biological Engineering, Laboratory of Research in Biofilms Rosário Oliveira, Minho University, Braga, Portugal; Northern Arizona University, UNITED STATES OF AMERICA

## Abstract

**Background:**

Bacterial vaginosis (BV) is a vaginal dysbiosis associated with adverse reproductive and infectious outcomes. Current diagnostics identify BV after symptom onset. We evaluated whether artificial neural network (ANN) models of the vaginal microbiome could predict incident BV (iBV) up to 14 days before clinical diagnosis.

**Methods:**

ANNs were trained using 16S rRNA gene sequencing data from 1,201 longitudinal vaginal specimens collected from 58 women across two prospective cohorts. Models classified individual specimens as pre-iBV or healthy using the relative abundance of vaginal bacterial taxa. Model performance was assessed using a held-out participant-level test set, participant-level cross-validation, and external validation. SHAP analysis was used to identify taxa associated with model predictions.

**Results:**

On the held-out participant-level test set, the ANN achieved 93% accuracy (AUC = 0.97, sensitivity = 95%, specificity = 92%). Models using only five taxa (*Lactobacillus crispatus*, *Gardnerella* spp., *L. iners*, *L. mulieris*, and *Megamonas* spp.) maintained >91% accuracy, sensitivity, and specificity. SHAP analysis identified *Lactobacillus* spp. and *Gardnerella* spp. as the taxa most strongly associated with model predictions. Participant-level cross-validation produced lower and more variable performance (AUC = 0.826 ± 0.076; accuracy = 71.5% ± 8.2%), while external validation achieved approximately 80% balanced accuracy.

**Interpretation:**

Vaginal microbiome composition contains predictive information that can identify women at risk of iBV before clinical onset. Lower performance during participant-level cross-validation and external validation indicates that additional validation in larger, more diverse cohorts is required before clinical implementation.

## Author summary

Background

Bacterial vaginosis (BV) is the most common vaginal infection worldwide and is associated with numerous adverse health outcomes. Changes in the vaginal microbiota occur 7–14 days prior to the onset of incident BV (iBV). Modeling vaginal microbiome compositional patterns leading up to iBV onset can be used to predict the development of iBV.

Key findings

In this study, we developed artificial neural network (ANN) models to predict iBV up to two weeks before clinical onset using the abundance of key vaginal bacterial taxa. The models achieved high performance on a held-out participant-level test set (>91% accuracy), while participant-level cross-validation provided a more conservative estimate of performance (72% accuracy; AUC = 0.83). Exploratory subgroup analyses also suggested that the importance of individual bacterial taxa may differ across demographic groups, highlighting the potential for personalized vaginal microbiome-based prediction.

Implications

Our models enable prediction of iBV by surveying the vaginal microbiome, serving as a valuable tool to determine which patients are at risk of developing iBV in time to intervene before iBV development. Prediction of iBV development could lead to wider adoption of clinical interventions useful in the prevention of iBV such as live biotherapeutics, prophylactic antibiotics, and/or behavioral modifications. Similarly, our findings highlight the value of developing models personalized to specific patient populations, improving accuracy while reducing the number of features required for predictions.

## Introduction

Bacterial vaginosis (BV) is the most common vaginal infection, affecting approximately 30% of reproductive-age women worldwide [1–3]. BV is a vaginal dysbiosis that is associated with multiple adverse health outcomes, including infertility, adverse birth outcomes, increased risk of HIV acquisition and other sexually transmitted infections (STIs), pelvic inflammatory disease, and increased risk of post-gynecologic surgery pelvic infections [1,4,5]. Although a large body of observational and interventional data suggest that BV is sexually transmitted [6–9], it remains unclear whether BV results from acquisition of a single key pathogen, a polymicrobial consortium of pathogenic bacteria, [2,10–12] or depletion of protective lactobacilli [13–16], which may allow for subsequent colonization by BV-associated bacteria (BVAB) [17].

The application of machine learning (ML) techniques to vaginal microbiome data presents an opportunity to advance BV diagnostics by modeling community dynamics prior to incident BV (iBV) [18]. Artificial neural networks (ANNs) are one such supervised ML tool that has been successfully applied to model complex microbial relationships [19], [20]. In our application of ANNs for vaginal microbiome analysis, sequencing-derived quantitative bacterial taxa data serve as ANN inputs [18]. This approach enables the identification of microbial signatures and patterns that may be critical in understanding and diagnosing BV.

In this study, we applied ANNs to predict iBV development using data from two prospective iBV pathogenesis studies [21–23]. While the prior studies tracked changes in vaginal microbial communities over time, our ANN models demonstrated predictive capability for identifying participants at increased risk of iBV within the next two weeks based on a single vaginal specimen. These models identify predictive microbial features associated with future iBV onset, support development of personalized diagnostics, and improve characterization of vaginal microbial taxa contributing to iBV pathogenesis.

## Results

### Clinical sampling and modeling workflow

For this study, we analyzed 1,201 self-collected vaginal specimens from a total of 58 women participating in 2 iBV pathogenesis studies (32 who developed iBV and 26 healthy controls) [21–23]. Following vaginal bacterial taxonomic assignment, 20 vaginal microbial taxa were retained for analysis after filtering to remove low-abundance, non-vaginal, or unclassified taxa to ensure biological relevance and model stability. These included *Lactobacillus iners, L. crispatus, L. mulieris, Gardnerella* spp.*, L. gasseri, Megasphaera lornae, Staphylococcus aureus, Finegoldia magna, Fannyhessea vaginae, Sneathia vaginalis, Aerococcus christensenii, Megamonas spp., Limosilactobacillus coleohominis, Prevotella timonensis, Corynebacterium kefirresidentii, Sneathia sanguinegens, Streptococcus anginosus, Candidatus Lachnocurva vaginae* (previously known as BVAB1)*, Gemella sanguinis,* and *Anaerococcus prevotii.*

**Fig 1:**
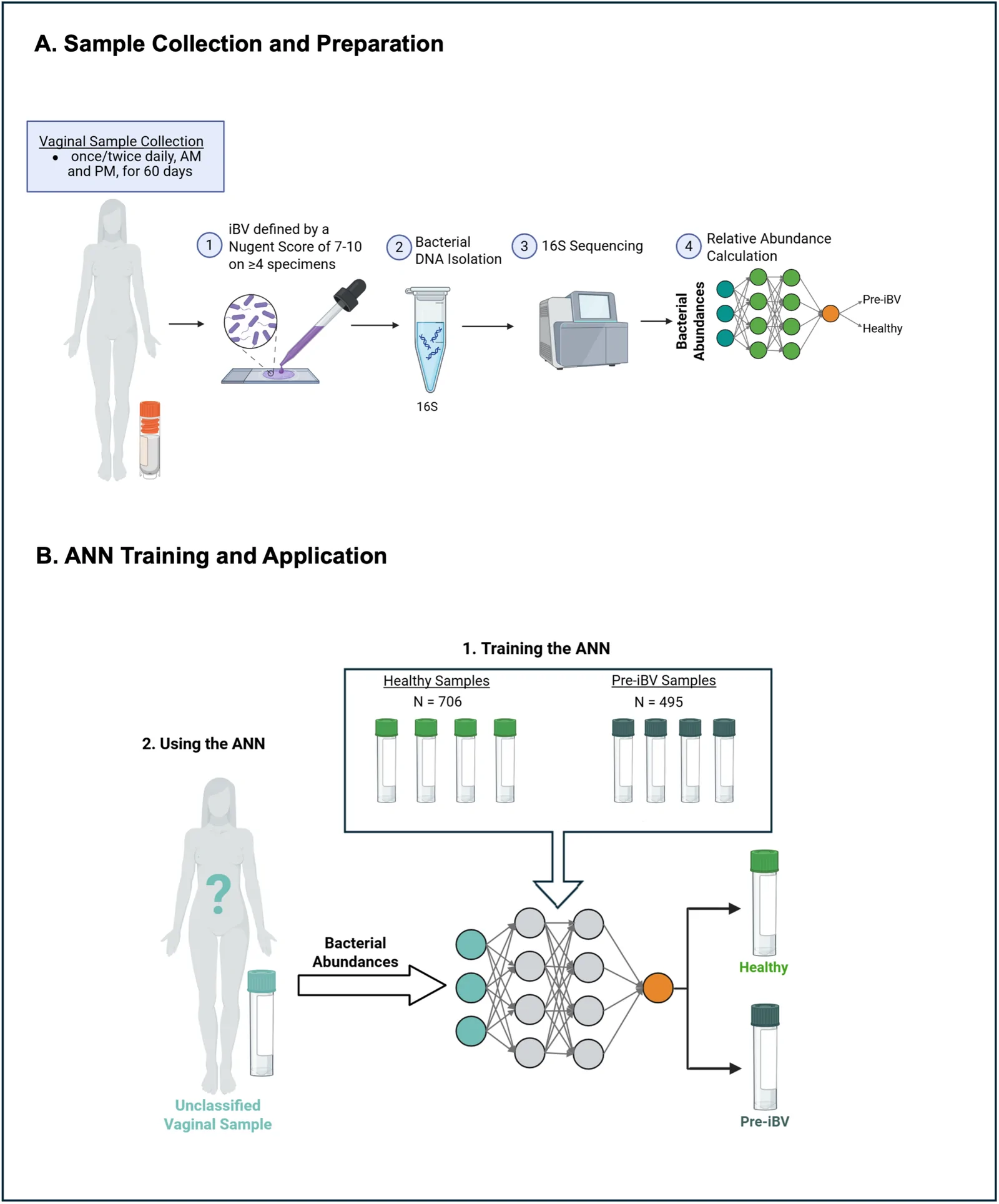
Study workflow for training ANNs to predict pre-iBV from vaginal specimens. (A) Daily vaginal specimens were collected from 58 participants and monitored for development of iBV using Nugent scoring. DNA was isolated from vaginal specimens which was used for 16S sequencing. The relative abundance of key vaginal taxa was utilized to train ANN models to classify specimens as pre-iBV or healthy. **(B)** ANN models were trained using relative abundance of key vaginal taxa from individual specimens (n = 1201) to predict if a single specimen was from a participant who developed iBV within 14 days (pre-iBV) or from a participant who did not develop iBV within the next 14 days (healthy). The model was designed so that it can be used to classify a single vaginal sample as healthy or pre-iBV (figure created with BioRender.com).

**Fig 2:**
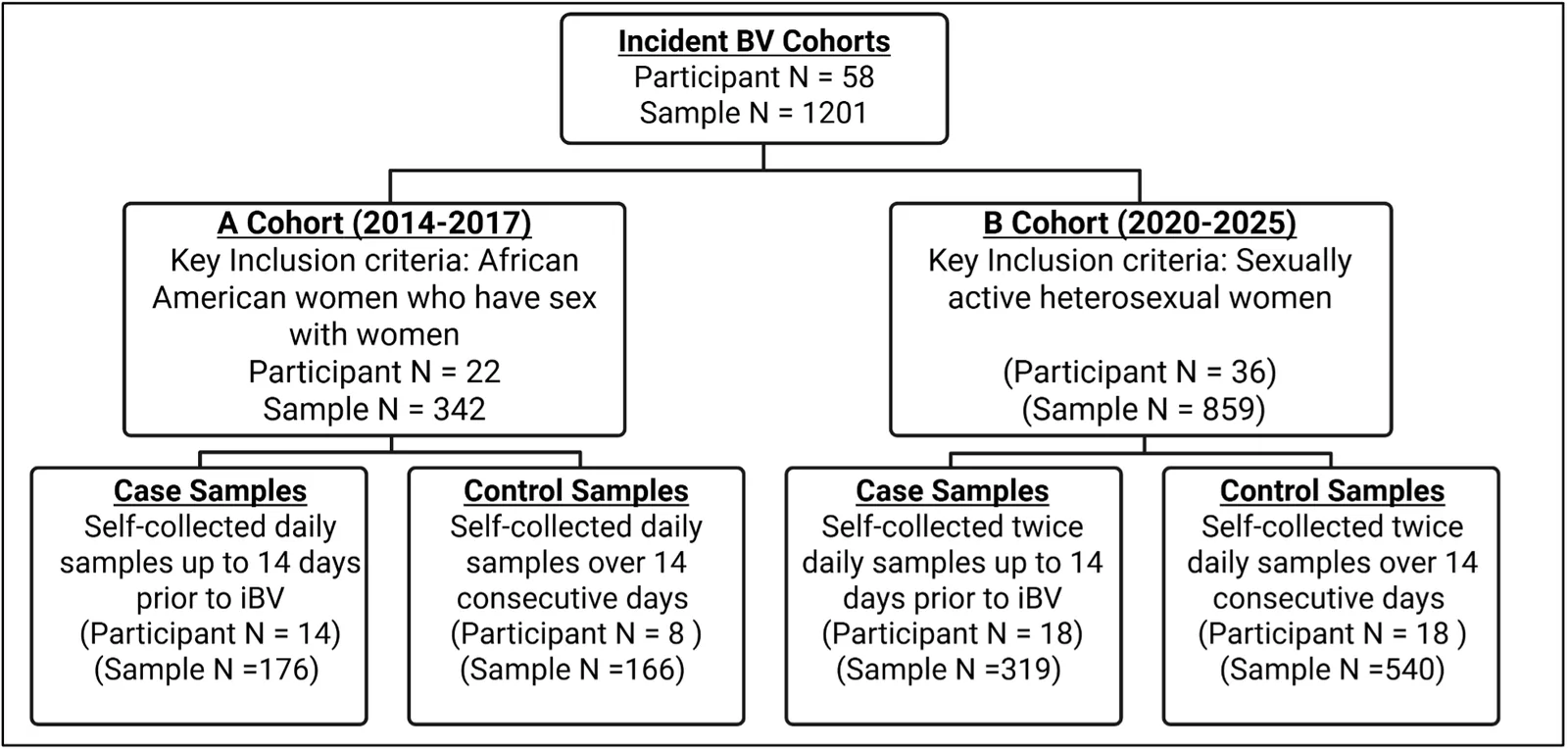
Incident bacterial vaginosis cohorts included in the study. Vaginal specimens collected from women participating in two longitudinal iBV pathogenesis studies were used to train the models. Cohort A consists of 22 African American women who have sex with women and Cohort B consists of 38 women who have sex with men between the ages of 18–40. Specimens in Cohort A were collected once daily during the 14 days leading up to iBV. Specimen in Cohort B were collected twice daily on the 14 days leading up to iBV.

Of these vaginal specimens, 495 were collected within 14 days prior to iBV onset (pre-iBV) and 706 were from healthy controls. The final ANN model was trained to predict whether a single vaginal specimen came from a pre-iBV participant or from a healthy participant. Cohort A consisted of African American women who have sex with women and included once-daily sampling, whereas Cohort B enrolled women who have sex with men from multiple racial backgrounds with twice-daily sampling. The workflow for specimen collection, data generation, and model training is shown in Fig 1, with cohort composition summarized in Fig 2.

### Modeling for early prediction of iBV

To assess whether characterization of the vaginal microbiota could aid in early prediction of iBV, we trained ANN models using the relative abundance of 20 vaginal bacterial taxa. Model performance was evaluated using multiple complementary approaches, including a held-out participant-level test set, participant-level cross-validation, and external validation analyses presented below. Because repeated specimens were collected from each participant, model evaluation was performed using participant-level data partitioning to prevent information leakage between training and testing datasets.

**Fig 3:**
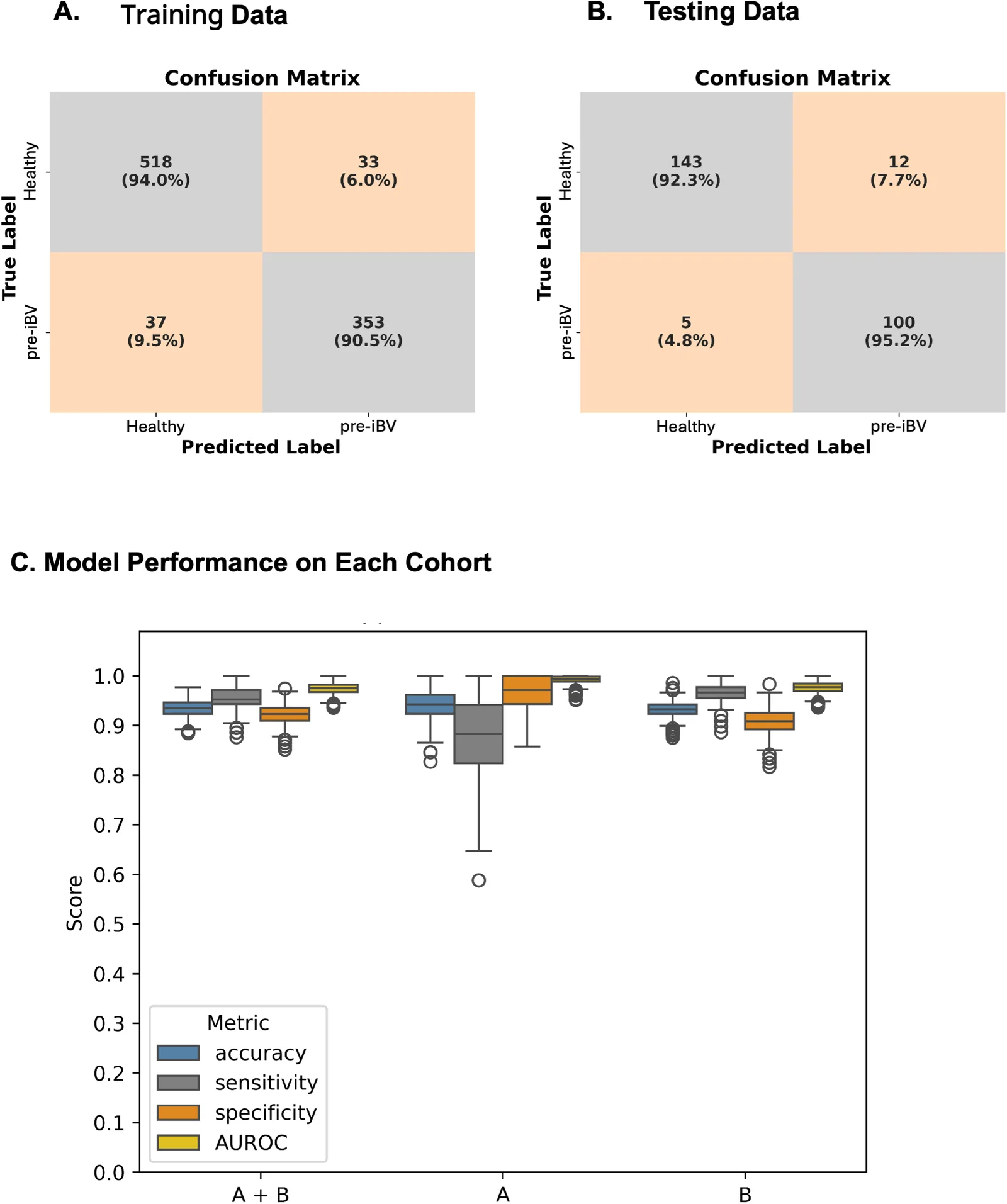
20-Feature Model Classification Performance & AUC. Summary of ANN modeling performance trained using 16S relative abundance data of 20 common vaginal bacterial taxa (n = 1201). (A-B) Confusion Matrices model classifications in relation to true classification of (A) training data (n = 941) and (B) testing data (n = 260). Tiles containing correct classifications are shown in grey and tiles containing incorrect classification are shown in orange. Tile labels specify the number of samples in each category and percentage of samples in each category based on their true classification. (C) Bootstrapped classification accuracy is shown for all samples combined, cohort A samples, and cohort B samples. Abbreviations: AUC = area under the curve.

On the held-out participant-level test set, the final ANN achieved 93% accuracy, 95% sensitivity, and 92% specificity (Fig 3, Table 1, Fig A in S1 Text). Vaginal specimens from the test set of Cohort A were classified with 94% mean accuracy, while specimens from the test set of Cohort B were classified with 93% mean accuracy (Fig 3C, Table 1). The model achieved a test AUC of 0.97 and Cohen’s kappa of 0.86. Final model performance was evaluated once on the held-out participant-level test set after hyperparameter selection (S8 Table in S1 Text).

**Table 1:** Model performance metrics. Classification accuracy on vaginal specimens used in the training set is shown in Train columns, while accuracy of model classification on testing samples is shown in Test columns. Metrics were calculated using data from both cohorts combined and for each separate cohort. Bootstrapping was used to calculate the mean values for each metric and with corresponding 95% confidence intervals, shown in each cell.

	A + B		A		B	
Metric	Train	Test	Train	Test	Train	Test
**Accuracy**	0.92 ± 0.03	0.93 ± 0.06	0.88 ± 0.03	0.94 ± 0.11	0.94 ± 0.03	0.93 ± 0.06
**Sensitivity**	0.90 ± 0.05	0.95 ± 0.08	0.86 ± 0.10	0.88 ± 0.29	0.93 ± 0.06	0.96 ± 0.07
**Specificity**	0.94 ± 0.03	0.92 ± 0.08	0.90 ± 0.09	0.97 ± 0.08	0.95 ± 0.04	0.90 ± 0.10
**F1 Score**	0.90 ± 0.04	0.92 ± 0.07	0.88 ± 0.06	0.90 ± 0.20	0.92 ± 0.04	0.92 ± 0.06
**AUC**	0.97 ± 0.01	0.97 ± 0.04	0.94 ± 0.05	0.99 ± 0.02	0.98 ± 0.01	0.97 ± 0.04
**PPV**	0.91 ± 0.05	0.89 ± 0.10	0.91 ± 0.07	0.94 ± 0.12	0.91 ± 0.06	0.88 ± 0.11
**NPV**	0.93 ± 0.03	0.96 ± 0.05	0.84 ± 0.10	0.94 ± 0.17	0.96 ± 0.03	0.97 ± 0.05
**Cohen. Kappa**	0.84 ± 0.07	0.86 ± 0.13	0.76 ± 0.13	0.86 ± 0.27	0.88 ± 0.07	0.86 ± 0.12

Abbreviations: AUC = area under the curve, PPV = positive predictive value, NPV = negative predictive value

Training performance was similar to held-out test performance, with a mean training accuracy of 92%, sensitivity of 90%, specificity of 94%, and AUC of 0.97 (Table 1). These findings indicate that the model effectively captured microbial patterns associated with future iBV development within the study population.

To evaluate model performance across the defined predictive window, we assessed classification accuracy separately for early pre-iBV specimens (6–14 days prior to iBV onset) and late pre-iBV specimens (1–5 days prior). The model correctly classified 97% of early pre-iBV specimens and 77% of late pre-iBV specimens across training and testing sets. Classification accuracy results are shown in **Fig B in** S1 Text, and metrics are listed Table B in S1 Text. Overall, these findings indicate that predictive performance varies across the predictive window, with higher accuracy observed at earlier timepoints prior to clinical onset.

### Participant-level cross-validation performance

To further evaluate model robustness and reduce dependence on a single train/test split, we performed participant-level three-fold cross-validation within the training dataset. All specimens from a given participant were restricted to a single fold to prevent within-participant data leakage.

Performance estimates obtained from participant-level cross-validation were substantially lower and more variable than those observed on the held-out participant-level test set, providing a more conservative estimate of model generalization. Model performance was assessed using AUC because it provides a threshold-independent measure of classification performance. The ANN achieved the highest mean AUC and accuracy across folds (AUC = 0.826 ± 0.076, accuracy = 71.5% ± 8.2%), followed by logistic regression (AUC = 0.769 ± 0.176, accuracy = 66.7% ± 13.7%) and random forest (AUC = 0.739 ± 0.127, accuracy = 64.3% ± 15.7%) (**Table F in** S1 Text). AUC distributions across folds are shown in **Fig I in** S1 Text.

These findings indicate that performance estimates derived from a single train/test split are likely optimistic relative to cross-validated performance. Nevertheless, all models retained predictive signal under participant-level cross-validation, with the ANN achieving the highest mean AUC, although differences between models were modest. Despite the more conservative performance estimates observed under cross-validation, the ANN was retained as the primary model for subsequent analyses because it consistently demonstrated strong performance across evaluation strategies and provides a flexible framework for modeling nonlinear relationships and higher-order interactions between vaginal microbial taxa.

To assess whether model performance was sensitive to the compositional structure of the vaginal microbiome data, we repeated the analysis using centered log-ratio (CLR)-transformed taxa features. Models were re-tuned and evaluated using the same participant-level framework and feature set (top 20 taxa) as in the primary analysis.

Under participant-level cross-validation within the training set, the ANN achieved the highest mean AUC (0.811 ± 0.100), followed by random forest (0.754 ± 0.127) and logistic regression (0.720 ± 0.212). However, on the held-out test set, logistic regression achieved the highest AUC (0.981), followed closely by random forest (0.979), while the ANN achieved an AUC of 0.914 **(Table G in** S1 Text).

Model performance metrics for the tuned CLR-based models are summarized in **Table G in** S1 Text. These findings indicate that predictive signal is preserved under CLR transformation and can be effectively captured across multiple modeling approaches. Similar trends were observed across both cross-validation and held-out test set evaluations. Under CLR-transformed features, logistic regression and random forest achieved performance comparable to the ANN on the held-out test set, supporting that the underlying predictive signal is robust to feature representation and model choice.

While CLR transformation provides a statistically appropriate representation of compositional data, relative abundance features were retained for the primary analysis for several reasons. First, relative abundance preserves direct biological interpretability, as values correspond to the proportional representation of taxa within the vaginal microbiome, consistent with standard reporting in microbiome and clinical literature [24]. In contrast, CLR-transformed values represent log-ratios relative to a geometric mean, which are less intuitive to interpret at the level of individual taxa.

Second, predictive performance was consistent across feature representations, and the primary conclusions of the study were not dependent on transformation choice. CLR transformation did not provide a consistent performance advantage for the ANN, and comparable performance was observed across models.

Finally, SHAP-based interpretation is more directly aligned with relative abundance features, allowing clearer attribution of model predictions to individual taxa. In CLR-transformed space, feature contributions are inherently relative to all other taxa, which complicates interpretation at the level of individual organisms. Accordingly, we retained relative abundance as the primary feature representation while presenting CLR-based results as a complementary sensitivity analysis.

### Model benchmarking across machine learning approaches

To assess model performance relative to simpler approaches and evaluate potential overfitting, we compared ANN, logistic regression, and random forest models trained using the same feature set (top 20 taxa) and identical participant-level data splits, ensuring direct comparability across models (**Table D in** S1 Text). All models were evaluated on a held-out participant-level test set.

All models demonstrated predictive capability on the held-out participant-level test set; however, the ANN achieved modestly higher performance in the primary relative-abundance analysis (**Table E in** S1 Text). Although differences in classification accuracy were modest (ANN: 0.977; logistic regression: 0.942; random forest: 0.931), the ANN reduced classification error rates by more than two-fold relative to comparator models. These findings suggest that simpler models capture much of the underlying microbial signal while the ANN provides modest additional predictive benefit.

### Role of microbial features in pre-iBV classification

**Fig 4:**
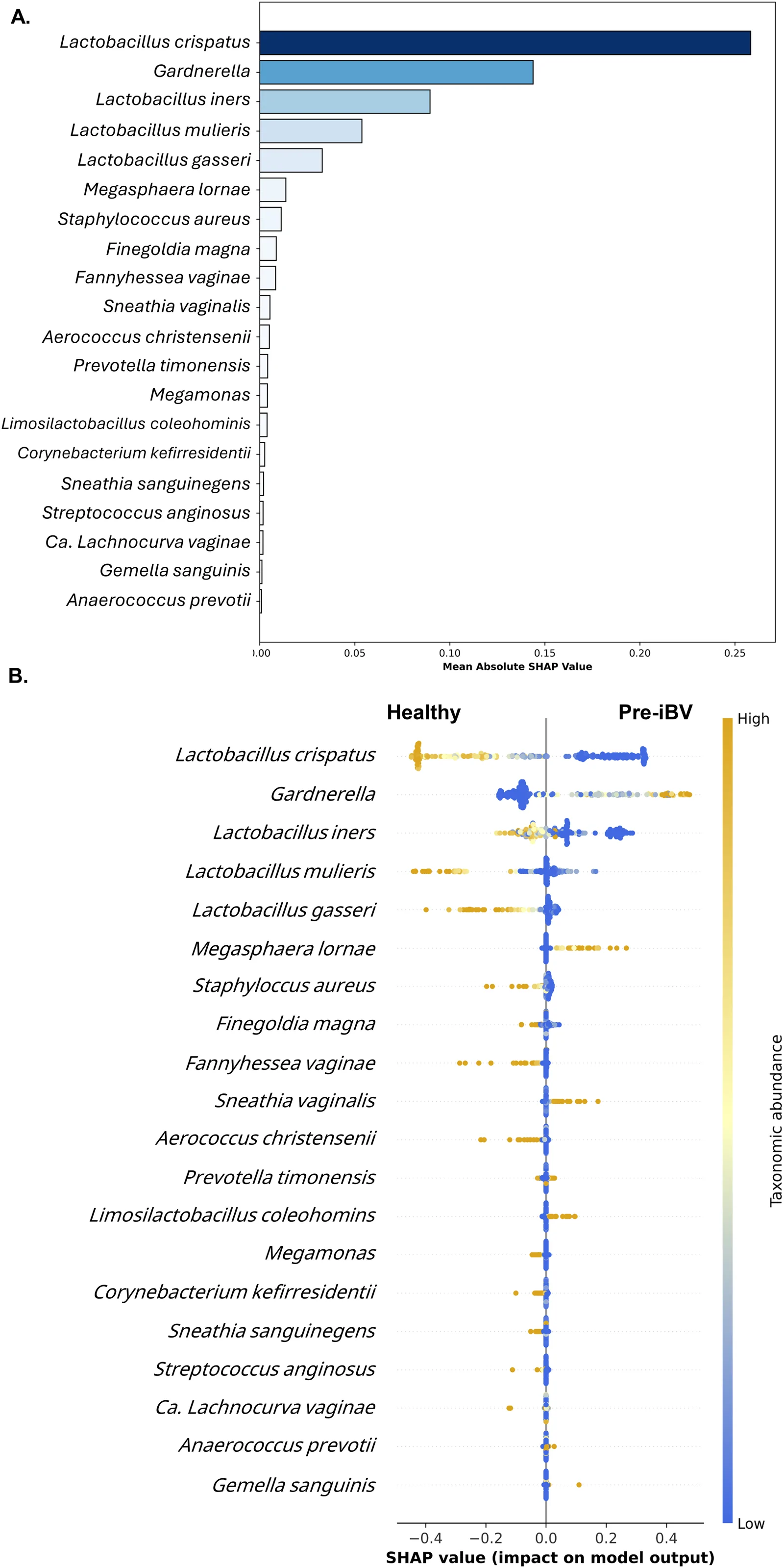
Feature importance and feature use determined from SHAP analysis. **(A)** Importance of features included in the 20-feature model determined by mean absolute SHAP value (n = 260). **(B)** Beeswarm plot visualizing how features contribute to model predictions. The relative abundance of each bacterial taxa is reflected by a color gradient: orange indicates a high relative abundance while blue indicates a low relative abundance. X-axis position indicates whether the feature contributes to predicting a vaginal specimen is classified as healthy (left) or pre-iBV (right). Distance from 0 on the x-axis indicates how much each feature contributes to accurately classifying an individual specimen as healthy or pre-iBV.

Shapely additive explanations (SHAP) analysis of the 20-feature model assessed how each taxon (feature) contributed to model predictions (Fig 4) [25]. Because the model was trained using relative abundance data, SHAP values should be interpreted as reflecting the contribution of bacterial taxa to model predictions within a compositional microbial community rather than as independent biological effects of individual organisms. This analysis revealed that several vaginal *Lactobacillus* spp. ranked highly as important features for model accuracy [26]. Specifically, higher relative abundance of *L. crispatus, L. iners, L. mulieris*, and *L. gasseri* was associated with model predictions favoring healthy classifications. Conversely, higher relative abundance of BV-associated taxa including *Gardnerella* spp. and *Megasphaera lornae* was associated with model predictions favoring pre-iBV classifications. Because relative abundance data are compositional, these associations reflect model behavior within the context of the overall vaginal microbial community and should not be interpreted as independent biological effects of individual taxa.

### Minimum feature analysis

**Fig 5:**
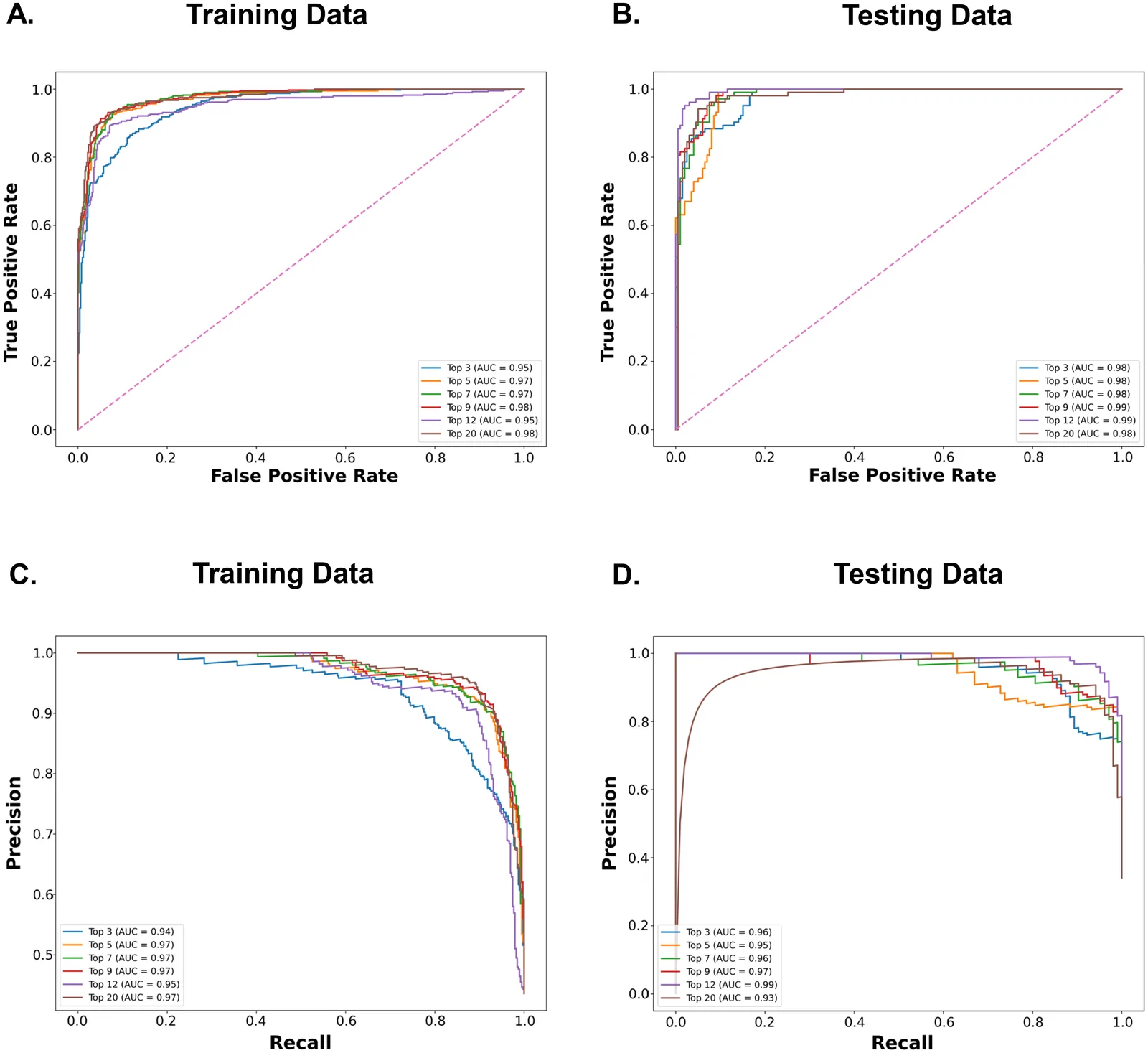
N-feature models receiver operating curves and precision-recall curves. Performance of models trained on the three, five, seven, nine, and twelve most important features determined by mean absolute SHAP value are visualized as **(A-B)** receiver operating curves and **(C-D)** precision-recall curves. **(A,C)** Curves calculated from training data (n = 941). **(B,D)** Curves calculated from testing data (n = 260).

To evaluate the number of vaginal bacterial taxa required to accurately classify specimens, successive models were constructed with a training strategy using a limited subset of features. Specifically, five subset models were generated through training models using the top 3, 5, 7, 9, and 12 most important features (Fig 5, Table 2, Fig C in S1 Text). The specific taxa included in each subset model are listed in Fig D in S1 Text. The model trained using the top three most important features (*L. crispatus, Gardnerella* spp.*, L. iners*) had a peak training accuracy of 87.6% and a testing accuracy of 89.6%. Models trained using the top 5, 7, 9, and 12 features achieved peak training accuracy ranging from 90-93% and peak sensitivity ranging from 91-97%, indicating that the inclusion of additional taxa improves overall performance and classification of pre-iBV specimens. These findings indicate that, while surveying a few key vaginal bacterial taxa is sufficient to classify specimens as pre-iBV or healthy, incorporating additional taxa enhances the classification of pre-iBV specimens. Across analyses, ANN, logistic regression, and random forest models demonstrated broadly comparable performance, indicating that multiple approaches capture meaningful microbiome signals. We retained the ANN as the primary model due to its ability to model nonlinear relationships and higher-order feature interactions, while noting that simpler models achieved broadly comparable performance. When models were evaluated separately by cohort, both Cohort A and Cohort B specimens were classified with high accuracy, though Cohort A specimens consistently showed higher classification performance across all subset models. (**Fig E in** S1 Text).

**Table 2:** N-feature model performance metrics. Classification accuracy metrics for models trained on the top three, five, seven, nine, and twelve most important features. Classification metrics of training and testing specimens are shown in Train columns and Test columns. Bootstrapping was used to calculate average values and 95% confidence interval, shown in each cell.

	Top 3		Top 5		Top 7		Top 9		Top 12	
Metric	Train	Test	Train	Test	Train	Test	Train	Test	Train	Test
**Accuracy**	0.87 ± 0.04	0.89 ± 0.07	0.92 ± 0.03	0.91 ± 0.06	0.92 ± 0.03	0.91 ± 0.06	0.93 ± 0.03	0.93 ± 0.05	0.90 ± 0.03	0.93 ± 0.05
**Sensitivity**	0.85 ± 0.06	0.97 ± 0.06	0.93 ± 0.04	0.91 ± 0.10	0.90 ± 0.05	0.93 ± 0.09	0.92 ± 0.05	0.96 ± 0.06	0.87 ± 0.06	0.90 ± 0.10
**Specificity**	0.88 ± 0.05	0.84 ± 0.12	0.91 ± 0.04	0.91 ± 0.08	0.93 ± 0.03	0.90 ± 0.06	0.93 ± 0.03	0.91 ± 0.09	0.92 ± 0.04	0.96 ± 0.05
**F1 Score**	0.85 ± 0.05	0.88 ± 0.07	0.91 ± 0.03	0.89 ± 0.08	0.90 ± 0.03	0.90 ± 0.07	0.91 ± 0.03	0.92 ± 0.06	0.88 ± 0.04	0.92 ± 0.07
**AUC**	0.95 ± 0.02	0.96 ± 0.04	0.97 ± 0.01	0.96 ± 0.04	0.97 ± 0.01	0.97 ± 0.03	0.98 ± 0.01	0.96 ± 0.03	0.96 ± 0.01	0.93 ± 0.08
**PPV**	0.84 ± 0.06	0.80 ± 0.11	0.88 ± 0.05	0.88 ± 0.10	0.91 ± 0.05	0.87 ± 0.10	0.90 ± 0.04	0.88 ± 0.10	0.89 ± 0.05	0.94 ± 0.08
**NPV**	0.89 ± 0.04	0.977 ± 0.04	0.95 ± 0.03	0.94 ± 0.06	0.93 ± 0.03	0.95 ± 0.06	0.94 ± 0.03	0.97 ± 0.04	0.91 ± 0.03	0.93 ± 0.06
**Cohen.Kappa**	0.74 ± 0.08	0.79 ± 0.14	0.84 ± 0.06	0.82 ± 0.14	0.84 ± 0.06	0.83 ± 0.13	0.85 ± 0.06	0.86 ± 0.11	0.80 ± 0.07	0.87 ± 0.12

Abbreviations: AUC = area under the curve, PPV = positive predictive value, NPV = negative predictive value

SHAP analysis was applied to determine how features were utilized by the n-feature models (**Fig F in** S1 Text). Across all n-feature models, *L. crispatus* and *Gardnerella* spp. consistently emerged as the two most important bacterial taxa, similar to findings from the 20-feature model. Overall, SHAP analysis indicates that *Lactobacillus* spp. and *Gardnerella* spp. are consistently strongly associated with model predictions across all feature sets tested. These findings should be interpreted as model-derived associations within a compositional microbial framework rather than evidence of independent biological effects or causal relationships.

### External validation performance

To assess generalizability beyond the original study population, models were evaluated using an independent external cohort consisting of 421 vaginal specimens from 27 participants. Performance declined relative to internal evaluation, with the 20-feature model achieving a balanced accuracy of 80% and AUC of 0.83. This reduction in performance is consistent with the more conservative estimates observed during participant-level cross-validation. This reduction in performance should be considered a limitation when interpreting the potential clinical utility of the model. Although predictive signal remained detectable in the external cohort, the decline in performance indicates that model behavior is sensitive to cohort-specific biological and methodological differences, highlighting the need for additional validation across independent cohorts before clinical implementation. This represents a meaningful reduction from internal performance (>93% accuracy on the held-out participant-level test set). While performance varied across subset models, the top performing models included the 5-feature model and 12-feature model, which achieved a balanced accuracy score of 76% and 79%, with corresponding AUC values of 80% and 83% respectively. Model performance metrics are further listed in Table 3. Additionally, model calibration was assessed by calculating brier score and expected calibration error for internal and external data sets, listed in Table I in S1 Text.

**Table 3:** Model performance metrics on external validation cohort. Classification accuracy metrics for models trained on the top three, five, seven, nine, twelve, and twenty features.

Metric	Top 3	Top 5	Top 7	Top 9	Top 12	Top 20
**Balanced Accuracy**	0.67	0.76	0.71	0.63	0.79	0.80
**Accuracy**	0.54	0.77	0.83	0.62	0.81	0.79
**Sensitivity**	0.83	0.74	0.58	0.64	0.77	0.80
**Specificity**	0.51	0.78	0.85	0.62	0.81	0.79
**AUC**	0.78	0.83	0.82	0.77	0.80	0.83
**Youden’s J**	0.35	0.52	0.43	0.27	0.58	0.60

Abbreviations: AUC = area under the curve

Notably, model performance in the external cohort was characterized by a reduction in balanced accuracy (from >93% internally to approximately 80% externally), with a relative shift toward higher sensitivity and reduced specificity, indicating a tendency to over-predict pre-iBV in this dataset. This likely reflects a combination of reduced generalizability and important differences between cohorts, including iBV case definition, vaginal specimen sampling frequency, geography, cohort demographics, and potential variation in sequencing or preprocessing workflows. Because the external cohort defined iBV based on a single Nugent score ≥7 rather than the stricter iBV definition used in the training cohorts (≥7 on ≥2 consecutive days), these results should be interpreted as evaluating performance on a related but distinct phenotype rather than a direct replication of the original prediction task.

To further investigate differences between cohorts, we compared the distributions of key taxa between the training and external cohorts stratified by BV status (**Fig J in** S1 Text; **Table J in** S1 Text). This analysis revealed cohort-dependent differences in microbial composition within disease states. In the BV/pre-iBV group, BV-associated taxa including *Gardnerella* spp. and *F. vaginae* exhibited higher relative abundance in the external cohort, while *S. vaginalis* also demonstrated higher relative abundance in the external cohort among BV/pre-iBV samples within BV states. In contrast, *L. iners* differed in its relative abundance between cohorts across both healthy and BV/pre-iBV groups, with changes in median abundance and overall distribution shape, suggesting differences in baseline community structure. *L. crispatus* remained comparatively stable across cohorts, particularly in the healthy group. Overall, these findings suggest that differences in microbiome structure between cohorts, in addition to differences in iBV case definition, likely contributed to reduced external model performance.

Statistical comparisons within BV strata are summarized in **Table** J **in** S1 Text and support significant differences for multiple taxa between cohorts, indicating that these shifts are not explained solely by differences in iBV case composition.

### Race-specific models: Performance and feature utilization

**Fig 6:**
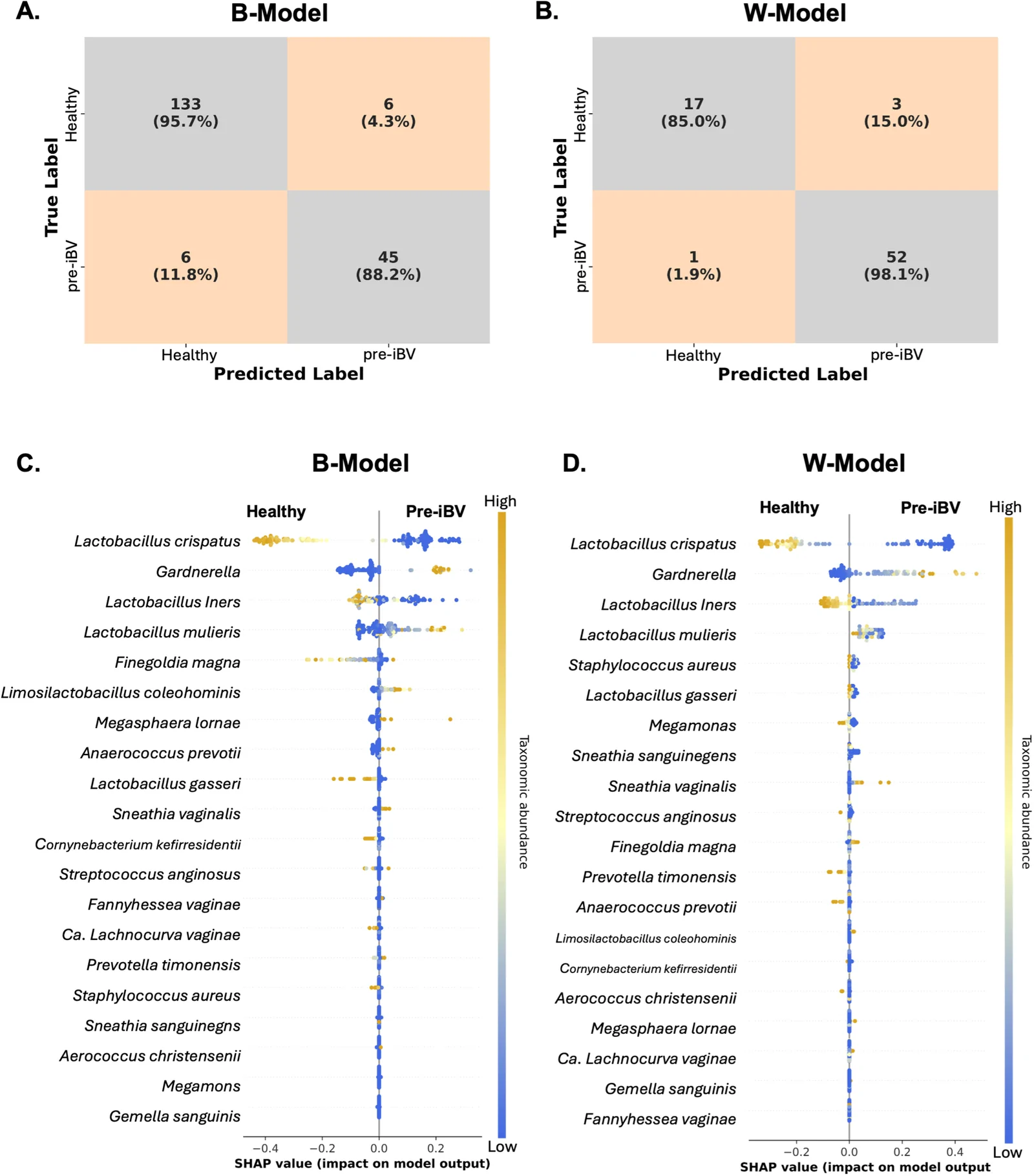
Classification performance and SHAP analysis of models trained on race-specific data. **(A-B)** Confusion matrices of classification results of testing data on models trained on specimens from **(A)** Black participants (n = 811) or **(B)** White participants (n = 380). Grey tiles indicate correct classifications by the model. Tile labels specify the number and percentage of specimens in each category based on their true classification. **(C-D)** Beeswarm plots indicate feature importance and how features contribute to model predictions. SHAP analysis was performed on testing data from models trained using specimens from **(C)** Black participants (n = 190 testing specimens) or **(D)** White participants (n = 73 testing specimens). The relative abundance of each taxa is reflected by the color gradient; orange indicates high relative abundance and blue indicates low relative abundance for a given taxa in comparison to other samples. X-axis position indicates whether the feature contributes to predicting a specimen is classified as healthy (left) or pre-iBV (right). Distance from 0 on the x-axis indicates how much each feature contributes to accurately classifying an individual specimen.

Considering there is evidence suggesting that the composition of the vaginal microbiota differs based on race, [24,27] we trained models separately using specimens from Black (B-Model, n = 811) and White (W-Model, n = 380) participants. Because these analyses were performed on substantially smaller participant subsets, particularly within the White subgroup, results should be considered exploratory and interpreted primarily as hypothesis-generating. After training, the W-Model achieved ≥94% accuracy on both the training and testing datasets, while the B-Model achieved ≥90% accuracy (Fig 6, Table B in S1 Text). The disparity in performance is likely due to the B-Model including data from both Cohort A and Cohort B as training and testing data, while the W-Model only utilized data from Cohort B.

**Fig 7:**
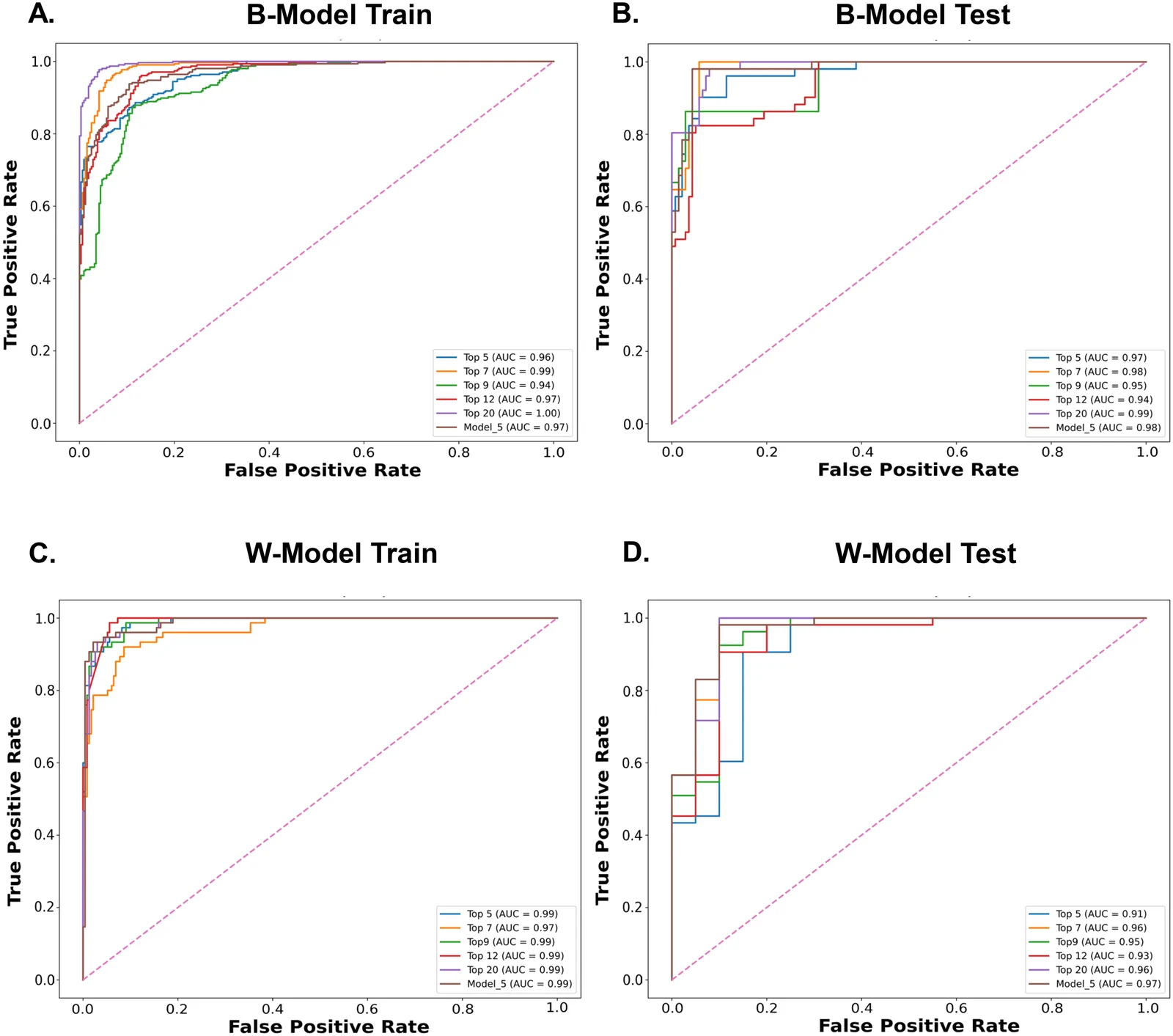
Receiver operating curves of race-specific n-feature models. **(A-B)** Receiver operating curves of models trained the top three, five, seven, nine, twelve most important features to B-model predictions based on classification on **(A)** training data (n = 572) and **(B)** testing data (n = 239). **(C-D)** Receiver operating curves of models trained the top three, five, seven, nine, and twelve most important features to W-Model predictions based on classification on **(C)** training data (n = 190) and **(D)** testing data (n = 73).

Race-specific models were additionally trained using feature subsets to evaluate whether high classification performance could be maintained using fewer features. Both models performed well when trained on only the three most important features. The B-Model achieved a test accuracy of 95% (AUC > 0.99), whereas the W-Model achieved a test accuracy of 88% (AUC = 0.96). Peak classification accuracy was observed for the 9-feature B-Model (92%) and the 7-feature W-Model (100%), although the latter was based on a relatively small participant subgroup (Fig 7, Table C in S1 Text).

To further evaluate how individual features contributed to model predictions, SHAP analysis was performed on each race-specific subset model (Figs G, H in S1 Text). Across both the Black (B-models) and White (W-models) participant models*, L. crispatus* and *Gardnerella* spp. consistently ranked as the most important features. A high relative abundance of *L. crispatus* was associated with the model predicting a healthy classification, while a high abundance of *Gardnerella* spp. was associated with the model predicting pre-iBV, consistent with findings from the 20-feature models. Interestingly, in the B-models, *L. iners* ranked as the third most important feature but did not show a clear relationship with either healthy or pre-iBV states. These exploratory results suggest that model performance and the relative contribution of individual bacterial taxa may differ across demographic subgroups.Larger and more diverse cohorts will be required to determine whether these observations are reproducible. As with the primary model, these SHAP-derived associations should be interpreted as reflecting model behavior within compositional microbiome data and not as independent biological effects of individual taxa.

The limited number of participants contributing to each subgroup, particularly in the White cohort (9 training, 5 testing participants), should be considered when interpreting these performance estimates, as repeated sampling within a small number of individuals may inflate apparent model stability (**Table H in** S1 Text).

## Discussion

In this study, we developed ANN models capable of predicting iBV up to 14 days prior to clinical onset using a single vaginal specimen. The models were trained using vaginal specimens from two distinct longitudinal cohorts of women with dense sampling and well-defined iBV endpoints [21–23]. While the held-out participant-level test set demonstrated high classification accuracy, participant-level cross-validation and external validation produced substantially lower performance estimates, indicating that model generalizability remains incomplete across independent populations [28]. From training models with a variety of taxa subsets, we found that an ANN model trained on 20 vaginal bacterial taxa achieved the highest overall classification performance across the evaluated feature sets. Participant-level cross-validation also demonstrated greater variability in performance, suggesting that estimates derived from a single train/test split are likely optimistic relative to expected performance in new participants. Despite these limitations, all evaluation strategies demonstrated meaningful predictive signal, supporting the potential utility of vaginal microbiome features for identifying individuals at increased risk of iBV. This predictive capability may enable earlier identification of individuals at risk for iBV and provide opportunities for proactive interventions to prevent clinical onset.

Notably, model performance was higher for specimens collected earlier within the predictive window (6–14 days prior to onset) compared to those collected closer to iBV diagnosis (1–5 days prior). This finding suggests that the model may be better at identifying early microbial changes associated with the initiation of dysbiosis rather than the immediate pre-diagnostic state. One possible explanation is that microbial communities in the earlier pre-iBV period reflect more consistent deviations from healthy baseline states, including loss of protective *Lactobacillus* spp. and increased abundance of key BV-associated taxa such as *Gardnerella* spp., consistent with current conceptual models of iBV pathogenesis.^12^ In contrast, specimens collected closer to diagnosis may represent more variable community compositions characterized by expansion of multiple BV-associated bacteria, which may reduce separability between groups. This reduced separability may limit model performance during the late pre-iBV phase. These findings have important implications for clinical application, suggesting that the model may be most effective for identifying individuals at elevated risk before symptom onset rather than detecting imminent iBV. These findings suggest that the actionable clinical window for intervention may lie earlier in the disease trajectory when microbial shifts are less complex and potentially more amenable to preventative strategies.

From a clinical perspective, these findings suggest that the intended use of the model may be for risk stratification rather than imminent diagnosis. Individuals identified as high risk during the earlier pre-iBV period could potentially be targeted for increased surveillance, behavioral interventions, live biotherapeutics, or other preventative strategies before progression to diagnosed iBV [29,30]. Accordingly, the primary clinical value of this approach may lie in the early identification of individuals at elevated risk to enable proactive interventions rather than detecting disease immediately before onset.

Feature analysis provided insight into microbial patterns associated with the model’s predictions of pre-iBV and healthy states. From SHAP analysis, we found that common vaginal *Lactobacillus* spp. and *Gardnerella* spp. were the taxa most strongly associated with accurate classification of specimens as healthy or pre-iBV [23,26,27]. As expected, higher abundance of *Lactobacillus* spp. was associated with the model predicting healthy classifications, while higher abundance of *Gardnerella* spp. was associated with the model predicting pre-iBV. These findings are consistent with current conceptual models of iBV pathogenesis, which describe early loss of protective *Lactobacillus* spp. and expansion of BV-associated taxa such as *Gardnerella* spp [10,31,32].

Additionally, our results support the notion that *L. crispatus* is critical to protection against iBV, while less common *Lactobacillus* spp., such as *L. gasseri* and *L. mulieris* (previously classified as *L. jensenii*), [33] may vary in their protective capacity due to strain variation and host features [34,35]. Additionally, we found that taxa in the genus *Megamonas* ranked high as the fifth most important feature in subset models. While this taxon has not previously been strongly implicated in iBV, its prominence in the model may reflect a role in metabolic interactions within dysbiotic communities, such as participation in short-chain fatty acid production or cross-feeding networks that support BV-associated bacteria. Further studies will be required to assess the relationship between *Megamonas* and iBV pathogenesis.

Importantly, these findings should be interpreted as model-derived associations rather than evidence of biological causality, as SHAP values reflect features that are important for model predictions rather than mechanistic relationships. However, the consistency between SHAP-identified taxa and known biological mechanisms in iBV pathogenesis strengthens the plausibility of these associations.

Consistent with current models of iBV pathogenesis, our findings support the hypothesis that colonization by *Gardnerella* spp. and depletion of protective *Lactobacillus* spp. occur as early events preceding iBV onset [12]. The strong contribution of *Gardnerella* spp. in SHAP analysis is consistent with its established role in initiating polymicrobial biofilm formation on the vaginal epithelium and producing virulence factors such as sialidase that degrade host mucus barriers and facilitate colonization by other BV-associated bacteria (secondary colonizers). These processes promote microbial adhesion, biofilm stability, and immune modulation, contributing to the transition from a *Lactobacillus*-dominant to a dysbiotic vaginal environment. In parallel, reduced abundance of *Lactobacillus* spp., particularly *L. crispatus*, reflects loss of lactic acid production and maintenance of low vaginal pH, which are critical for suppressing the growth of BV-associated anaerobes. Together, these findings demonstrate that the taxa identified as most influential by SHAP analysis correspond to key biological mechanisms underlying BV pathogenesis [17,27].

Minimum feature analysis revealed that only a few taxa were necessary for accurate predictions. Models trained on the five most important features (*L. crispatus, Gardnerella* spp., *L. iners, L. mulieris, and Megamonas*) achieved >90% accuracy. While the inclusion of additional vaginal bacterial taxa slightly improved accuracy, the strong performance of minimal models suggests that simplified approaches to characterizing the vaginal microbiota may be sufficient for specific research and diagnostic purposes. For example, using targeted qPCR panels of five to seven organisms could be feasible alternatives to complex sequencing-based profiling for early iBV prediction.

Exploratory race-stratified analyses demonstrated differences in model performance relative to the combined-cohort model; however, these findings are based on small participant numbers and should be interpreted cautiously. These findings are consistent with prior studies showing that longitudinal changes in vaginal bacterial community composition differ by race [24,27]. While the overall predictive features were similar between groups, *L. crispatus* and *Gardnerella* spp. consistently ranked as the strongest predictors. In contrast, the contribution of *L. iners* differed notably. In models trained on specimens from Black participants, *L. iners* was variably associated with both healthy and pre-iBV states, suggesting its dual role as a transitional species within less stable microbial communities. In contrast, in models derived from White participants, *L. iners* was more often associated with healthy classifications, consistent with greater community stability. These findings underscore that race-specific microbial ecology influences how key bacterial taxa relate to vaginal health and dysbiosis, highlighting the possibility that subgroup-specific modeling approaches may warrant future investigation, although these findings should be interpreted as exploratory and require validation in larger, more diverse, independent cohorts.

Race should not be interpreted as a strictly biological variable, but rather as a proxy for a complex combination of social, environmental, behavioral, and biological factors that influence the vaginal microbiome [36]. Although the top predictive taxa were consistent between groups, the relative contribution of each varied by race, suggesting that the microbial context surrounding shared taxa differs across populations. These findings highlight the importance of considering host demographic and microbial diversity in model development, as aggregating data across heterogeneous groups may obscure population-specific microbial patterns relevant to iBV pathogenesis and diagnostic accuracy.

Beyond the core predictive taxa incorporated into our final models, several additional bacterial features emerged as highly ranked during feature selection, despite not being retained in the optimized ANN models. These taxa, including *F. vaginae*, *P. bivia*, *M. lornae,* and *Sneathia* spp*.*, have been implicated in iBV pathogenesis and recurrence through their roles in biofilm formation, metabolic cross-feeding, and host immune modulation [10,37]. The inclusion of these taxa in our top performing classifiers supports current models of BV as a polymicrobial infection characterized by synergistic interactions among multiple BVAB rather than dominance by a single bacterial pathogen. Moreover, the identification of these taxa reinforces the biological relevance of our modeling approach by independently highlighting microbial players known to contribute to vaginal dysbiosis. These findings also have potential clinical implications, as early prediction of iBV could allow for early clinical interventions such as live biotherapeutics, prophylactic antibiotics, and/or behavioral modifications to prevent development of iBV.

While we have rigorously conducted our analysis and added valuable insight into the early prediction of iBV, there are several limitations to our study. An important consideration is the potential for overfitting given the relatively small number of participants and the large number of model configurations evaluated. To address this, we implemented participant-level cross-validation and directly compared ANN performance to logistic regression and random forest models using identical data splits and feature sets. Performance estimates were lower under cross-validation, providing a more conservative estimate of generalization; however, all models retained predictive signal, and the ANN demonstrated a modest advantage in mean AUC. These findings indicate that model performance is robust across modeling approaches, while emphasizing that single train/test split estimates may overstate real-world performance. Another limitation is that we did not incorporate additional clinical or behavioral metadata into the predictive models. Our primary goal was to determine whether microbiome composition alone could predict iBV onset, but inclusion of variables such as menstrual status, contraceptive use, sexual activity, and other host factors may improve predictive performance and generalizability in broader clinical settings. Future studies should evaluate multimodal modeling approaches that integrate microbiome, clinical, behavioral, and other host-derived data.

This study also relies on taxonomic (16S rRNA gene) data without incorporation of functional measurements such as metabolomics or transcriptomics. As a result, while specific taxa were identified as important for model predictions, the underlying biological mechanisms driving these associations cannot be directly assessed. Integration of functional multi-omics data will be critical in future studies.

An additional limitation is that microbiome relative abundance data are compositional by nature, meaning taxa are constrained by a unit-sum structure and are therefore not statistically independent. This property can influence both model behavior and interpretation of SHAP-derived feature importance. To address this, we performed a complementary analysis using centered log-ratio (CLR)-transformed features and found that predictive signal was largely preserved across representations. Under CLR transformation, logistic regression and random forest achieved performance comparable to the ANN, while ANN performance was modestly reduced. These findings indicate that model performance is robust to feature representation and that predictive signal is not dependent on transformation choice.

Despite the statistical advantages of CLR transformation, we retained relative abundance as the primary feature representation for interpretability. Relative abundance aligns with standard reporting in microbiome research and allows direct interpretation of taxa contributions. In contrast, CLR-transformed features represent relative log-ratios, which complicate interpretation at the level of individual taxa. Accordingly, SHAP results should be interpreted as model-based associations rather than independent or causal effects of individual taxa.

Another limitation of this study is the relatively small cohort size (n = 58 women), which may limit generalizability and contribute to variability in participant-level performance estimates. Additionally, we used the Nugent score as the reference standard for defining iBV. While widely used, Nugent scoring is based on morphological assessment of several bacterial morphotypes on vaginal Gram stain and does not fully capture the complexity, functional activity, or temporal dynamics of the vaginal microbiome. As a result, it may not perfectly align with underlying microbial states relevant to disease progression or model prediction.

Additionally, the data used to train our ANN models combined two cohorts with sampling biases. Cohort A exclusively included African American women, while Cohort B reflects the racial demographics of the Birmingham, AL metropolitan area. These sampling biases limit our ability to identify relationships for other racial and ethnic groups such as Hispanics, Asians, and Native Americans [36,38,39]. Future studies validating our models in more diverse cohorts will be required.

A limitation of this study is the reduction in performance observed during external validation. Balanced accuracy decreased from >93% in the held-out participant-level test set to approximately 80% in the external cohort. While predictive signal remained detectable, this decline indicates that model performance does not fully generalize across independent cohorts and is sensitive to both biological and methodological differences between studies. Consequently, performance estimates derived from the original cohorts likely overstate performance in new settings. Additionally, predictions were well calibrated internally but overestimated pre-iBV risk in the external cohort, potentially due to lower pre-iBV prevalence. As with the performance reduction described above, this reinforces that the models are best suited for relative risk stratification rather than absolute risk estimation, and that recalibration to the target population would be required before clinical use.

Several important differences between datasets likely contributed to this reduction. The external cohort differed in geographic location, sampling frequency, participant characteristics, and iBV case definition. Most notably, iBV in the external cohort was defined as a single Nugent score ≥7, [27] whereas the training cohorts required sustained Nugent score elevations across consecutive timepoints. Consequently, the external validation cohort represents a related but not identical prediction task rather than a direct replication of the original study design. Supporting this interpretation, supplementary analyses demonstrated cohort-specific differences in vaginal microbiome composition within both healthy and BV-associated states (**Fig J in** S1 Text; **Table J in** S1 Text). Additional contributors may include differences in sequencing workflows, preprocessing pipelines, and other unmeasured cohort characteristics.

Taken together, these findings indicate that broader clinical deployment cannot be inferred from the current validation results alone. Future studies should evaluate model performance using prospectively collected cohorts with harmonized study designs, microbiome profiling methods, and outcome definitions to more accurately assess generalizability.

Regardless of these limitations, our models offer an opportunity to enhance clinical diagnostic platforms by enabling early prediction of iBV in asymptomatic or pre-symptomatic women. Importantly, the current model generates predictions at the specimen-level rather than the participant-level. In a potential clinical implementation, longitudinal specimen-level predictions would likely be aggregated across time to estimate participant-level risk. For example, repeated positive classifications across serial vaginal specimens could be used to identify individuals with sustained elevated risk, whereas isolated positive predictions may warrant continued monitoring rather than immediate intervention. Future studies should evaluate participant-level prediction strategies and determine optimal approaches for integrating repeated microbiome measurements into clinically actionable risk estimates. This represents an advance beyond current FDA-cleared BV molecular diagnostics, which are exclusively authorized for use in symptomatic women only (Aptima BV Assay, Hologic, Marlborough, MA; Xpert Xpress MVP, Cepheid, Berkeley, CA; BD MAX Vaginal Panel, Geneohm, Québec City, QC) [40]. By identifying early microbial shifts predictive of BV, clinicians could intervene prior to disease onset and potentially reduce complications such as preterm birth or recurrent BV in certain populations [41].

Incorporating models such as those described in this study into the diagnostic development pipelines also holds promise for advancing personalized medicine in the context of microbiome-associated and polymicrobial diseases. Such approaches may be further strengthened by incorporation of relevant clinical and behavioral metadata alongside microbial features. Additionally, the use of these models, trained on population-specific data, can support the rapid identification of clinically relevant microbial targets to inform the design of diagnostic panels. This is particularly important given that existing FDA-cleared BV molecular diagnostics differ significantly in the microbial targets they include, with little overlap across platforms [40]. Integrating ANN approaches could standardize and optimize target selection, enabling the development of more inclusive and effective diagnostics that better reflect the diverse microbial populations associated with BV.

In summary, machine learning approaches identified predictive microbiome signals preceding iBV onset. While held-out test set performance was high, participant-level cross-validation and external validation produced more conservative estimates of performance, highlighting the need for additional validation in larger independent cohorts before clinical implementation. Importantly, external validation demonstrated a substantial reduction in performance relative to internal testing, indicating that model generalizability across populations remains incomplete and that broader clinical deployment cannot yet be supported. Our findings provide preliminary evidence that model performance and microbial predictors of pre-iBV may differ across demographic subgroups within this cohort, although these observations require validation in larger and more diverse populations. Because the present study evaluated prediction at the specimen-level rather than the participant-level, future work should determine how repeated specimen-level predictions can be aggregated to support participant-level clinical decision-making and risk stratification. Furthermore, these results support the hypothesis that iBV is mediated by a depletion of *Lactobacillus* spp*.* and enrichment of *Gardnerella* spp. prior to iBV onset. Early prediction of iBV could enable identification of individuals before clinical onset, when preventative interventions may be most effective, potentially allowing preventative interventions such as live biotherapeutics, prophylactic antibiotics, and behavioral modifications before progression to iBV [29,30]. Integrating personalized microbiome-based diagnostics capable of early prediction of vaginal disorders could revolutionize the management of BV and other important vaginal health conditions.

## Methods

### Ethics statement

This study was approved by the University of Alabama at Birmingham Institutional Review Board (UAB IRB; Protocol # IRB-300004547) with a reliance agreement at Louisiana State University Health Sciences Center New Orleans (LSUHSC New Orleans IRB; Protocol # 8738), as well as University of Alabama at Birmingham IRB Protocol # IRB-F131127001 [21,22]. Written informed consent was obtained for all participants in Cohorts A and B prior to enrollment. All vaginal specimens and participant data were de-identified prior to use in this project.

### Clinical enrollment

The training data were derived from two prospective longitudinal cohorts designed to study the sequence of microbiological events prior to iBV [21,23]. The two cohorts were active between 2014–2017 (Cohort A) [23] and 2020–2024 (Cohort B) [21], enrolling English-speaking women aged 18–45 in the Birmingham, AL metropolitan area.

Exclusion criteria for both cohorts included oral or intravaginal antibiotics within the past 14 days, self-reported HIV infection, or current pregnancy. Participants had to be asymptomatic at enrollment, with no Amsel criteria and normal Nugent scores of 0–3 with no *Gardnerella* spp. morphotypes on baseline vaginal Gram stain [42, 43]. At enrollment, participants in both cohorts were screened for *Trichomonas vaginalis*, *Chlamydia trachomatis*, *Neisseria gonorrhoeae* by highly sensitive nucleic acid amplification tests (NAATs). Participants who tested positive for one or more of these infections were treated per standard of care and dropped from the study [44]. A total of 58 participants were included in this ANN analysis: Cohort A included 22 participants, while Cohort B included 36 participants. Cohort A exclusively enrolled African American/Black women who have sex with women (WSW), while Cohort B enrolled women who have sex with men (WSM) of all races and ethnicities. Detailed enrollment criteria are available in prior publications [21–23].

Participants in Cohort A self-collected vaginal specimens once daily for 90 days or until the onset of iBV, while participants in Cohort B self-collected vaginal specimens twice daily for 60 days or until the onset of iBV. Additional details regarding specimen collection, delivery to the study site, storage, and preparation for 16S rRNA gene sequencing are available in prior publications [21,23]. These cohorts were specifically selected for this analysis due to their highly similar study designs and strict inclusion and exclusion criteria, which allowed for reliable detection of iBV cases with matching controls. Controls were participants that did not develop iBV during the course of the study and were matched to cases based on age, race, and contraceptive method. In addition, key non-microbiome factors relevant to iBV risk, including menses, contraception, and sexual activity, were accounted for through cohort design and specimen selection rather than included as model inputs.

### Selection of vaginal specimens for vaginal microbiome characterization

Participants were monitored for iBV throughout the course of both studies. iBV was defined as a Nugent score of 7–10 on ≥2 consecutive days in Cohort A and on ≥4 consecutive vaginal specimens/2 days in Cohort B and A. For women who developed iBV in both cohorts, specimens collected up to 14 days prior to iBV were selected for 16S sequencing. This 14-day interval defines the predictive window in this study, representing the time between microbiome sampling and subsequent iBV onset [43]. Within this predictive window, specimens were further categorized based on proximity to iBV onset to distinguish between earlier risk states (6–14 days prior) and more immediate pre-diagnostic states (1–5 days prior). This design enables evaluation of model performance across clinically relevant timeframes for early prediction versus near-term detection. This distinction was incorporated to evaluate whether microbiome-based prediction may be more informative for identifying earlier risk states suitable for preventative intervention versus imminent pre-diagnostic states immediately preceding clinical onset. Controls from both cohorts were selected if they maintained optimal vaginal microbiota for the majority of the study (e.g., a Nugent score of 0–3 for at least 85% of study days). Control specimens were matched to iBV case specimens by day of menses, as previously described [22,23]. All specimens were shipped to the Microbial Genomics Resource Group (MGRG) at the Louisiana State University Health Sciences Center (LSUHSC) in New Orleans, LA for DNA isolation, 16S rRNA sequencing, and bioinformatics analysis.

### Molecular methods

DNA isolated from stored vaginal specimens from women in both cohorts was used for 16S rRNA gene sequencing from women who developed iBV and matched healthy controls to determine changes in the relative abundance of vaginal bacteria over time.

DNA was isolated from the vaginal specimens, as described previously, using a modified QIAamp DNA Mini Kit (QIAGEN) [23,45]. PCR amplicon libraries spanning the taxonomically informative fourth hypervariable (V4) region of the 16S rRNA were generated and sequenced on the Illumina MiSeq platform [45]. Sequencing data were analyzed in R v4.2.1 using DADA2 v1.16 to identify sequence variants, [46] with taxonomic classification performed using SpeciateIT and SpeciateDB [47].

### External validation cohort

To ensure that the ANN models generalized beyond the original study populations and were not overfit to cohort-specific characteristics, we included an external validation cohort in this study to independently assess model performance on a distinct dataset. Validation data were obtained from PRJNA46331 (SRR17141071-SRR17141708) [27]. This external validation cohort consists of longitudinal vaginal specimens collected twice-weekly from 32 women living in the Baltimore, Maryland metropolitan area between 2008–2011, with accompanying Nugent scores and 16S rRNA sequencing data. Additionally, the external cohort differs from the training cohorts in geographic location (Baltimore, MD vs. Birmingham, AL) and sampling schedule (twice-weekly vs. daily or twice-daily sampling), which may influence observed microbiome dynamics and model performance. For the purposes of our study, iBV cases in the external cohort were defined as participants with a single Nugent score ≥7 at any time point (n = 12), and controls were defined as participants without a Nugent score ≥7 at any point during the study (n = 15). This definition differs from the stricter incident BV (iBV) definition used in the training cohorts, which required sustained Nugent score elevations across consecutive timepoints. As a result, the external validation task reflects a related but not identical prediction target. Vaginal specimens (n = 32) collected within 14 days prior the first Nugent score ≥7 were considered pre-iBV specimens; 389 specimens were used from the control population. Five participants were excluded due to missing Nugent scores or Nugent scores ≥7 at the first available timepoint. Amplicon sequence variants (ASV) were identified using the DADA2 workflow, and taxa were mapped to ASVs using SpeciateIT [47].

### Modeling approach and parameters

Using the sequencing data obtained from cohort A and B vaginal specimens, ANNs were built and compared using TensorFlow (v2.6.1) and Keras (v3.0) [48,49]. Specimens were grouped into “pre-iBV” (collected ≤14 days prior to iBV onset) or “healthy” (no iBV). The unit of prediction in this study was an individual specimen; however, all data partitioning was performed at the participant level to account for repeated sampling within individuals. Accordingly, all reported performance metrics reflect specimen-level classification performance rather than participant-level risk prediction. Using GroupShuffleSplit (scikit-learn) with participant ID as the grouping variable, the dataset was divided into a training set (80% of participants; n = 46) and a held-out test set (20% of participants; n = 12). This corresponded to 944 specimens in the training set and 257 specimens in the test set (**Table D in** S1 Text). All specimens from a given participant were assigned exclusively to either the training or test set, preventing information leakage and inflation of performance estimates due to within-participant correlation. The held-out test set was reserved exclusively for final model evaluation and was not used during model development. Model inputs included microbial taxonomic abundance. Clinical and behavioral factors such as menses, contraception, and sexual activity were addressed during cohort design, specimen selection, and were not included as predictors in models.

Models were trained and evaluated using two workflows, one using a three-fold cross validation approach, and then a second workflow was repeated using only a single training and testing split. For the cross-validation approach, hyperparameter tuning and model selection were performed using participant-level cross-validation (GroupKFold), ensuring that no participant contributed specimens to both training and validation folds. A predefined grid search was used to evaluate combinations of architecture and training parameters, including number of hidden layers, node combinations, dropout rates, Gaussian noise, batch size, and L1/L2 regularization strength. Mean cross-validated AUC was used as the primary model selection metric. For ANN training, an additional internal validation split derived solely from the training data was used for early stopping to prevent overfitting during final model fitting. To evaluate potential overfitting and benchmark model performance, we compared ANN models to logistic regression and random forest classifiers using identical data splits and feature inputs (top 20 taxa). All models were trained and evaluated using the same participant-level training and held-out test sets described above. Hyperparameters were selected based on mean cross-validated area under the receiver operating characteristic curve (AUC), which are listed in **Table K in** S1 Text.

To assess whether model performance was sensitive to the compositional nature of microbiome relative abundance data, we performed a complementary analysis using centered log-ratio (CLR)-transformed taxa features using the same cross-validation workflow. CLR transformation was applied to the same top 20 taxa used in the primary analysis after addition of a small pseudocount to accommodate zero values. This analysis was designed as a sensitivity analysis to evaluate robustness of model performance to feature representation rather than to replace the primary modeling framework.

Complementing the cross-validation approach, training was repeated on the complete participant-level training and testing sets to further assess modeling capabilities. A grid search approach was used to determine the optimal number of hidden layers, node combinations, dropout percentages, batch size, and regularization. In the second stage, the best-performing configuration was refined through manual tuning of gaussian noise and learning rate to produce the final model. Grid search parameters and final model parameters are listed in **Table L** in S1 Text. ANNs were implemented in Python3 in Visual Studio Code (v1.96.2). Visualizations were created using matplotlib (v3.7.1) [50].

For race-stratified analyses, datasets were subset by participant self-identified race prior to model training. Participant-level splits were then applied independently within each subgroup. Due to the smaller number of participants within each subgroup, particularly in the White cohort, these analyses were performed on limited participant samples. Participant- and sample-level distributions for race-specific train/test splits are provided in S8 Table in S1 Text. The Black subgroup included 35 participants (725 specimens) in training and 7 participants (86 specimens) in testing, while the White subgroup included 9 participants (257 specimens) in training and 5 participants (123 specimens) in testing. These differences reflect both the smaller number of participants in the White subgroup and the presence of repeated longitudinal sampling within individuals.

### Determination of key BV-associated bacteria in the development of iBV

Once the ANN models were trained and validated, we deconstructed them using the SHapley Additive exPlanations (SHAP) (v0.46.0) Kernel Explainer method to determine the “relative importance” of features included in each model [25]. SHAP provides a model-agnostic framework for interpreting complex non-linear models, such as ANNs, by estimating the marginal contribution of each feature across all possible feature combinations. This allows for both global feature importance and specimen-level interpretability. The training dataset was used as the background distribution, while testing data were used to compute SHAP values for individual predictions. To improve computational efficiency, training specimens were summarized into 50 representative samples using the shap.kmeans function. Mean absolute SHAP values were calculated for each feature by averaging SHAP values across all participants to assess global feature importance. SHAP values were also examined at the individual specimen-level to evaluate how features were associated with model predictions. These feature importance rankings were used to guide selection of reduced feature sets for subsequent models.

### Sample size estimation

Sample size of the cohorts was determined based on convenience sampling. All vaginal specimen data available at the time of analysis were included in the study.

### Statistical analysis and performance metric calculations

A bootstrapping approach was used to assess model performance metrics. For each model, 1000 bootstrap resamples were generated at the participant level rather than the specimen-level, such that all specimens from a given participant were retained together within each resampled dataset. Bootstrap resamples were used to calculate 95% confidence intervals and average model performance. Models were evaluated on training and testing sets using accuracy, Area Under Curve (AUC), Negative Predictive Value (NPV), Positive Predictive Value (PPV), sensitivity, specificity, F1 Score, and Kappa Cohen. Considering the external validation set was highly imbalanced (< 20% of specimens were pre-iBV) NPV, PPV, F1 score, and Kappa Cohen were not used to evaluate the models’ classification performance on external validation data. Balanced accuracy was included as an additional metric for evaluating external validation data classification performance since it equally weighed classification accuracy of healthy and pre-iBV specimens. Similarly, Youden’s J Index was included as an additional metric to summarize model performance on external validation specimens since it is insensitive to class imbalances. Model calibration was assessed by calculating brier score and external calibration error. Metrics were calculated using sci-kit learn (v1.6.1).

## Supporting information

S1 Text Fig A. **20-feature model training and validation curves.**Training and validation curves plotting (A) model loss calculated from binary cross entropy and (B) classification accuracy for 600 epochs of model training (training samples, n = 941; validation, n = 260). **Fig B. 20-feature model performance predicting early and late pre-iBV.**Accuracy of the 20-feature model was assessed for samples collected 6–14 days prior to iBV (early pre-iBV) and samples collected 1–5 days prior to iBV (late pre-iBV). **Fig C. N-feature model training and validation curves.**Training and validation curves plotting model loss over 400–600 epochs of training are shown on the left of each panel. Training and validation curves plotting accuracy over the course of training are shown on the right of each panel. **Fig D. Taxa used to train race-specific feature subset models.**Taxa included in each subset model as features are denoted with a check mark. **Fig E. Subset model performance stratified by cohort.** Metrics for each subset model were calculated for the grouped cohorts (A + B), Cohort A, and Cohort B. Each box plot was generated using accuracies from 1000 bootstrapped resamples of the testing data. **Fig F. SHAP analysis of n-feature models.** (A–E) Beeswarm plots indicating feature importance and feature contribution for models trained using the top three, five, seven, nine, and twelve most important taxa for model predictions. Orange indicates high relative abundance, and blue indicates low relative abundance. X-axis position indicates whether the feature contributes to predicting a sample is classified as healthy (left) or pre-iBV (right). Distance from 0 on the x-axis indicates the magnitude of each feature’s contribution to predictions. **Fig G. SHAP analysis of models trained on top feature sets in Black participants.** (A–E) Beeswarm plots indicating feature importance and feature contribution for models trained on samples from Black participants using the top three, five, seven, nine, and twelve most important taxa for model predictions. Orange indicates high relative abundance, and blue indicates low relative abundance. X-axis position indicates whether the feature contributes to predicting a sample is classified as healthy (left) or pre-iBV (right). Distance from 0 on the x-axis indicates the magnitude of each feature’s contribution to predictions. **Fig H. SHAP analysis of models trained on top feature sets in White participants.**(A–E) Beeswarm plots indicating feature importance and feature contribution for models trained on samples from White participants using the top three, five, seven, nine, and twelve most important taxa for model predictions. Orange indicates high relative abundance, and blue indicates low relative abundance. X-axis position indicates whether the feature contributes to predicting a sample is classified as healthy (left) or pre-iBV (right). Distance from 0 on the x-axis indicates the magnitude of each feature’s contribution to predictions. **Fig I. Distribution of AUC values across participant-level 3-fold cross-validation.** Distribution of AUC values across participant-level 3-fold cross-validation for the best-tuned ANN, logistic regression, and random forest models. Hyperparameters were selected within the training set using GroupKFold cross-validation. **Fig J. Distributions of key taxa in internal and external cohorts.** Boxplots show median and interquartile range on a log scale. Points represent non-zero observations. P-values from Mann–Whitney U tests are shown. Prevalence indicates proportion of samples with non-zero abundance.Tables **Table A. Classification accuracy of early and late pre-iBV samples.** Classification accuracy of the 20-feature model on late pre-iBV samples (≤5 days prior to iBV) and early pre-iBV samples (>5 days prior to iBV). Values shown indicate mean bootstrapped accuracy ± 95% confidence interval. **Table B. Modeling classification performance metrics for race-specific models.** Metrics for classification accuracy for models trained and tested only on samples from participants that self-identified as Black (B-model) or White (W-model). Training and testing metrics are shown. Bootstrapping was used to calculate average values and 95% confidence intervals.**Table C. Performance of race-specific models trained on feature subsets.** Metrics for classification accuracy for models trained on three, five, seven, nine, and twelve of the most important features from the 20-feature model for (A) Black participants and (B) White participants. Bootstrapping was used to calculate average values and 95% confidence intervals.**Table D. Participant- and sample-level distribution for the primary participant-level train/test split (seed = 1234).Table. Model benchmarking (primary split).Table E. Participant-level cross-validation performance (mean ± SD).Table F. Model performance using CLR-transformed taxa (tuned models).Table G. Participant- and sample-level distribution across race-specific train and test splits.Table H. Model calibration metrics on internal data splits and external data.Table I. Comparison of taxa distributions between internal and external cohorts, stratified by BV status.Table J. Hyperparameter Tuning of ANN Selected by Cross ValidationTable K. Neural Network Final Model Hyperparameter Tuning.**
(PPTX)
